# Addressing an HIV cure in LMIC

**DOI:** 10.1186/s12977-021-00565-1

**Published:** 2021-08-03

**Authors:** Sherazaan D. Ismail, Joshua Pankrac, Emmanuel Ndashimye, Jessica L. Prodger, Melissa-Rose Abrahams, Jamie F. S. Mann, Andrew D. Redd, Eric J. Arts

**Affiliations:** 1 Division of Medical Virology, Department of Pathology, Institute of Infectious Disease and Molecular Medicine, University of Cape Town, Cape Town, 7925 South Africa; 2 Department of Microbiology and Immunology, Schulich School of Medicine and Dentistry, University of Western Ontario, London, ON N6A5C1 Canada; 3 Center for AIDS Research Uganda Laboratories, Joint Clinical Research Centre, Kampala, Uganda; 4 Department of Epidemiology and Biostatistics, Schulich School of Medicine and Dentistry, University of Western Ontario, London, ON N6A 5C1 Canada; 5 Division of Infectious Diseases, Department of Medicine, Johns Hopkins University School of Medicine, Baltimore, MD USA; 6 Laboratory of Immunoregulation, Division of Intramural Research, National Institute of Allergy and Infectious Diseases, NIH, Bethesda, MD USA; 7 Bristol Veterinary School, University of Bristol, Langford House, Langford, Bristol, BS40 5DU UK; 8 Division of Infectious Diseases, Department of Medicine, Case Western Reserve University, Cleveland, OH 44106 USA

**Keywords:** HIV-1, Cure, Reservoir, Low-and-middle income countries, LMICs

## Abstract

HIV-1 persists in infected individuals despite years of antiretroviral therapy (ART), due to the formation of a stable and long-lived latent viral reservoir. Early ART can reduce the latent reservoir and is associated with post-treatment control in people living with HIV (PLWH). However, even in post-treatment controllers, ART cessation after a period of time inevitably results in rebound of plasma viraemia, thus lifelong treatment for viral suppression is indicated. Due to the difficulties of sustained life-long treatment in the millions of PLWH worldwide, a cure is undeniably necessary. This requires an in-depth understanding of reservoir formation and dynamics. Differences exist in treatment guidelines and accessibility to treatment as well as social stigma between low- and-middle income countries (LMICs) and high-income countries. In addition, demographic differences exist in PLWH from different geographical regions such as infecting viral subtype and host genetics, which can contribute to differences in the viral reservoir between different populations. Here, we review topics relevant to HIV-1 cure research in LMICs, with a focus on sub-Saharan Africa, the region of the world bearing the greatest burden of HIV-1. We present a summary of ART in LMICs, highlighting challenges that may be experienced in implementing a HIV-1 cure therapeutic. Furthermore, we discuss current research on the HIV-1 latent reservoir in different populations, highlighting research in LMIC and gaps in the research that may facilitate a global cure. Finally, we discuss current experimental cure strategies in the context of their potential application in LMICs.

## Introduction

The advent of antiretroviral therapy (ART) has converted the HIV-associated death sentence into a lifelong, manageable illness for those with adequate access. However, for many low-and middle-income countries (LMICs), access to sustained ART for the full population is challenging due to a variety of socio-economic factors. This is especially true in regions with the greatest infection burden, Eastern and Southern Africa, which account for more than half of all the people living with HIV-1 (PLWH). While, globally, more PLWH are aware of their seropositive status (~ 80%) and are accessing treatment (~ 67%) [1], this still falls short of the original 90-90-90 goal set forth by the United Nations for awareness of status, accessing treatment, and viral suppression, respectively. In addition, due to the development of drug-resistant strains, the ART failure rate for PLWH on first-line regimens is 5% per year, necessitating constant development of new treatments over time. Lifelong ART also imposes a substantial financial burden on already-constrained public health systems; as PLWH continue to live longer, the overall cost of ART has risen to an estimated 26.2B USD globally in 2020 [1] and could reach 40B USD if goals for 2030 are going to be met. Even a long-term remission of HIV-1 disease [undetectable viral load (VL)] in the absence of ART, as opposed to a sterilizing cure, would save the world from millions of future deaths and trillions of USD in drug and health care costs. Finally, even with full viral suppression there is still a high risk for other long-term morbidities, including an increased risk of heart, bone, and kidney disease [2, 3], and PLWH can be impacted socially, financially, and psychologically due to HIV stigma and discrimination. Thus, there is an urgent need for a cure for HIV-1.

**Fig. 1:**
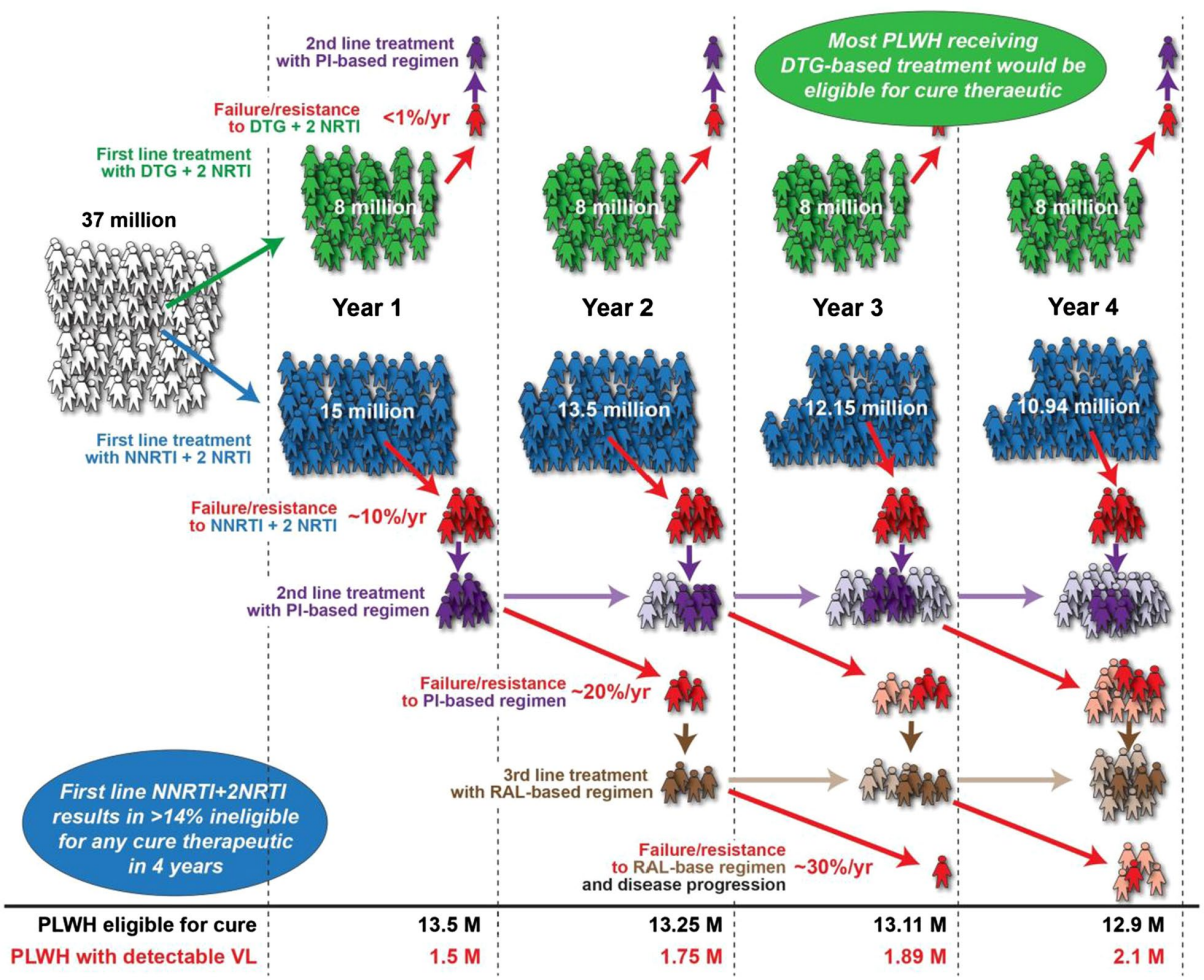
ART regimen chosen for first-line therapy can affect eligibility for a therapeutic cure. The incidence of treatment failure and/or drug resistance on an NNRTI+2 NRTI regimen is greatly increased relative to individuals receiving DTG+2 NRTI treatment. As a result of failing first-line therapy, PLWH may initiate PI- and RAL-based regimens, which have heightened incidence of failure and resistance. Effective ART allows more PLWH to maintain first-line therapy and facilitates initiatives to achieve therapeutic cure. *NNRTI* non-nucleoside reverse transcriptase inhibitor, *NRTI* nucleoside reverse-transcriptase inhibitor, *DTG* dolutregravir, *PI* protease inhibitor, *RAL* raltegravir

The benefits of an HIV-1 cure to PLWH and the public-health system in LMICs are clear and undeniable (Fig. 1). However, as discussed below, most cure approaches in preclinical development, or even those tested in clinical trials, may be financially prohibitive and impractical to administer in the near future in many LMICs, especially in sub-Saharan Africa. Currently, even just participation in clinical trials testing cure modalities requires frequent VL monitoring, reservoir quantification (currently developed only for subtype B), and full viral suppression, all readily available in high income countries (HICs) [4], but not in LMICs. Universally, the major barrier for HIV-1 cure is the existence of a long-lived latent reservoir consisting of stably integrated proviruses that persist in the host within various cell types and anatomical locations. Proviruses within the reservoir capable of reactivation and re-establishing plasma viraemia upon ART cessation persist despite years of suppressive ART [5–7]. PLWH exhibit differences in infecting viral subtype, immune responses to HIV-1, and disease progression, notably between individuals in LMICs vs. HICs, as well as between sexes [8–10]. Similarly, differences exist in the size, composition, and turnover in the reservoir between PLWH.

In this review, we discuss the viral and host cellular phenomena contributing to HIV-1 proviral latency establishment and persistence. With this knowledge, we will discuss the most relevant strategies for intervention and cure, highlighting scientific gaps that may influence generalizability of findings to LMICs.

## The current state of ART in LMICs

Early ART has been shown to limit seeding of the HIV-1 reservoir and is associated with post-treatment control. By diminishing the amount of immune activation, CD4^+^ T cell depletion and VLs in untreated infection, what results is fewer target cells for HIV-1 infection, preservation of the immune response [11–13], and a lower burden of viraemia over time. Thus, effective HIV-1 treatment in LMICs is necessary to implement a cure therapeutic. This may require use of more potent ART as a functional cure strategy during early stage of HIV infection. However, challenges of limited early HIV diagnosis, and access to potent ART regimens in LMICs need to be addressed. Successful distribution of generic DTG in LMICs through the Medicines Patent Pool (MPP) program [14] coupled with high rate of adherence by patients in LMICs [15] are good indicators that a cure therapeutic in LMIC setting can be achieved. Furthermore, optimization of a cure approach that will be effective in the millions of PLWH who initiated treatment during chronic infection is imperative. Therefore, to establish the potential utility of cure strategies in LMICs, it is essential to understand differences in ART treatment rollout among diverse global populations.

Global access to ART has increased tremendously. By end of 2019, over 26 million people had access to ART of which 17.9 million (69%) were PLWH in sub-Saharan Africa [16]. Despite this staggering number, the proportion of PLWH on ART in sub-Saharan Africa is still well below the UN target of 90%, and this is also true for South America (62%) and Asia and the Pacific (60%). Historically, first-line regimens in sub-Saharan Africa have included two nucleoside reverse transcriptase inhibitors (NRTIs) and one non-nucleoside reverse transcriptase inhibitor (NNRTI), commonly efavirenz (EFV) or nevirapine [17]. However, due to the combination of frequent drug supply/access issues in LMICs resulting in treatment interruptions [18] and the low genetic barrier to HIV-1 drug resistance mutations associated with NRTI + NNRTI regimens [19], 10–15% of patients who start ART still fail within one year, and 70–80% of people with virological failure develop acquired drug resistance [20]. In Kampala, Uganda, failure of first-line treatment continues to occur at an annual rate of 9% [21]. This statistic of treatment failure and drug resistance is rarely mentioned in discussion of the 90-90-90 goals by various global enterprises championing these goals.

For patients failing first-line therapy, the guidelines in most sub-Saharan African countries [and supported by the Global Fund and the U.S. President’s Emergency Plan for AIDS Relief (PEPFAR)] calls for a second-line ART of an NRTI + protease inhibitor (PI) and, subsequent to the failure of second-line, a third-line four-drug therapy of the NNRTI etravirine, the PI darunavir, and an integrase inhibitor with any suitable NRTI [22–24]. The choice of “salvage” treatment regimens in LMICs is limited due to lack of access to CCR5 antagonists, fusion inhibitors, and second-generation PIs and NNRTIs- agents with a higher barrier to HIV-1 drug resistance employed when patients fail treatment with first-generation PIs and NNRTIs. Limited ART options may exacerbate problems facing ART programs in sub-Saharan Africa, including adherence on ART, which directly translates to increased mortality. Indeed, a recent modelling study shows that 6 months of ART disruption for 50% of people would result in 296,000 more AIDS-related deaths in sub-Saharan Africa over one year [18]. Furthermore, frequent use of suboptimal NRTI + NNRTIs regimens has led to a > 10% prevalence of strains resistant to either NNRTIs or NRTIs in treatment-naïve PLWH [25]. Without pre-screening for drug resistance prior to initiation of first-line ART, treatment failure is likely to increase over time. High rates of treatment interruption and/or first-line failure present a huge barrier to cure therapeutic testing in sub-Saharan Africa, as all protocols to date have required participants to be fully virally suppressed. However, this need not be a barrier; provided there is stable drug supply, effective distribution to clinics/pharmacies, and ease of access to PLWH, adherence and success of first-line treatment is outstanding and often better in LMICs than HICs.

Despite the sombre predictions described above, the roll out of a dolutegravir (DTG)-based regimen (TDF+3TC/FTC+DTG) as a preferred first-line treatment at the end of 2017 has slowly improved the treatment success rates in all LMICs. Current estimates indicate that between 5 and 10 million PLWH in sub-Saharan African countries and other LMICs are receiving a DTG-based regimen [26]. In treatment naïve individuals, DTG-based treatment regimens are extremely well tolerated with minimal adverse events promoting high treatment adherence. Treatment failure and resistance to DTG is extremely rare in clinical studies to date due in part to high genetic barrier for resistance [27] to these second-generation integrase inhibitors (INSTIs). If the combination of DTG, bictegravir and the long-acting cabotegravir were available in dual or triple drug formulations, especially as one-pill-a-day, treatment success would improve dramatically in LMICs and thus provide a population of PLWH in LMICs that could be treated with an affordable and practical cure therapeutic.

Treatment outcome studies in Africa and other LMICs are heavily affected by different sociodemographic factors between countries, regions, ethnic groups, tribes, and religions [28–31]. As previously described in several cross sectional and longitudinal analyses on treatment adherence and HIV-1 drug resistance in Uganda and other sub-Saharan African countries, one of the greatest contributors to treatment failure is poor access to care/ARVs and intermittent ARV shortages, even more so than differential sociodemographic factors. Across sub-Saharan Africa and in LMICs during COVID-19 pandemic, ARV shortages, disruption in shipments, and reduced foreign aid will likely contribute to ART failure by orders of magnitude higher than the impact of HIV-1 types/subtypes, human genetics, and sociodemographic factors.

**Fig. 2:**
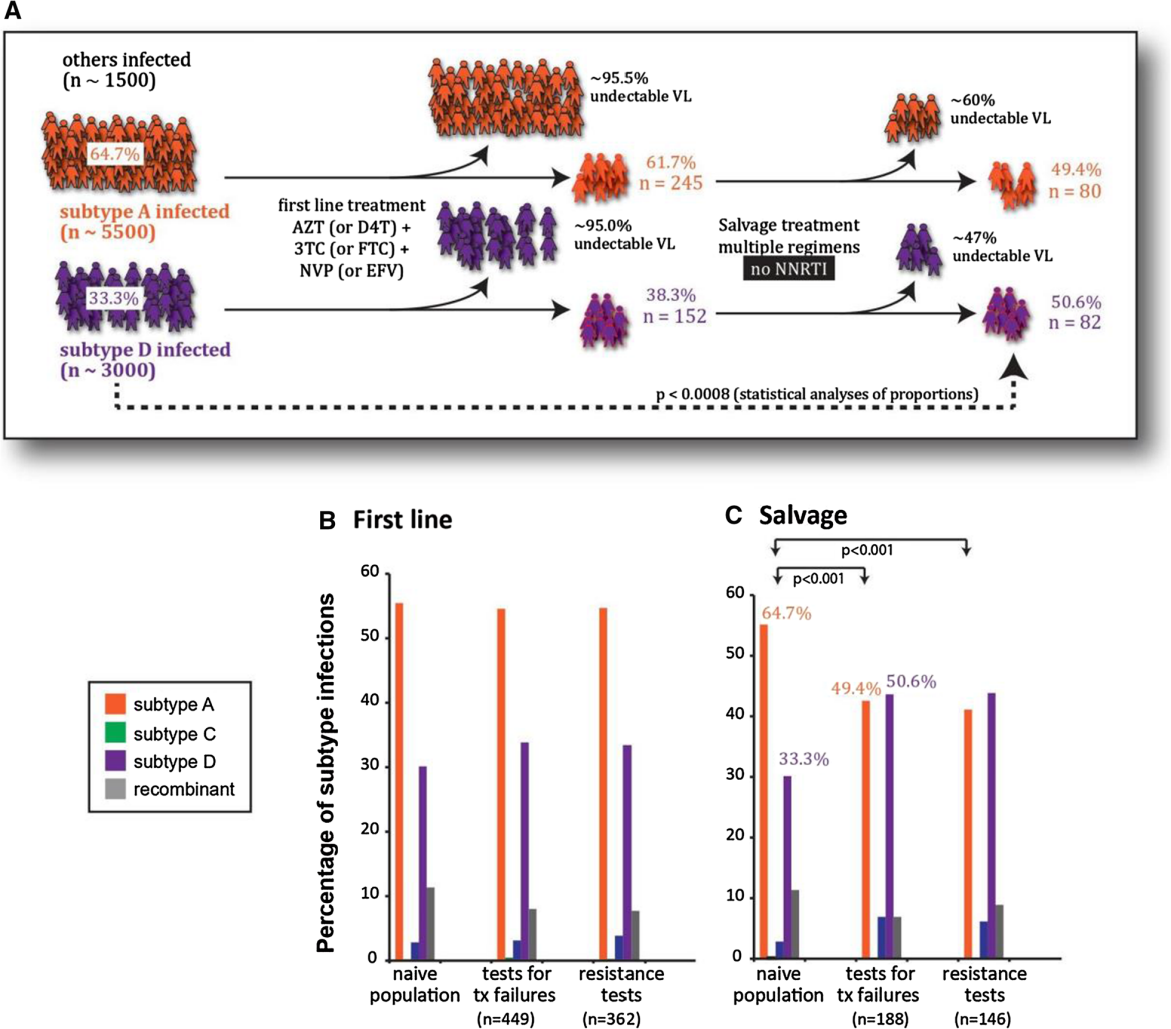
Faster subtype D versus subtype A HIV-1 treatment failure observed over first- and second-line treatments. The Joint Clinical Research Centre in Kampala, Uganda follows approximately 12,000 HIV-infected patients treated with antiretroviral drugs. Resistance testing is performed in cases of treatment failure—VL > 1000 copies/ml or two CD4 cell counts < 200/ml. ~ 95% of all patients on first-line treatment maintain undetectable VL in Uganda. < 50% reach and maintain undetectable VL on salvage therapy. As part of standard of care, we subtype (C) and analyze drug resistance genotypes of all patients failing treatment (D) on first-line (E) or “salvage” therapy (F)

In a specific area where the HIV-infected population are impoverished and share similar sociodemographic conditions, religion, ethnicity, etc., the co-circulating HIV-1 subtypes and/or CRFs could impact the effectiveness of any proposed cure therapeutic. Like all antiretroviral drugs developed to date, preclinical development and early phase human clinical trials of cure therapeutics have all been based on the ability to inhibit HIV-1 subtype B isolates, i.e., the HIV-1 strains that predominate in HICs. While ARTs have similar inhibitory effectiveness regardless HIV-1 subtype in terms of initial response to treatment [32–35], the effect of subtype on the emergence of drug resistance is not yet fully understood. For instance, despite similar in vitro susceptibility of subtype C and B strain-derived HIV-1 integrase enzymes to the currently-approved INSTIs, differential levels of drug resistance were observed between HIV-1 subtype B versus C viruses in cell culture assays, likely resulting from differential mutational pathways being favored between different subtypes [36]. Additionally, in vivo development of drug DTG resistance occurs at a slower rate among PLWH infected with subtype B compared to subtypes A/G and C [37]. Drug resistance to NRTIs and PIs has been shown to occur more frequent in subtype B compared to subtype C (26% vs. 8% for NRTIs, and 54 vs. 23% for PI’s, respectively) [38]. In Uganda, drug resistance and treatment failure is more prevalent in subtype D compared to A or C-infected patients (Fig. 2) [39]. These differences could be explained by variability of HIV-1 genes at the amino acid level between different subtypes and higher entropy scores observed at sites where drug resistance mutations emerge [40].

Differences in disease progression exist depending on infecting viral subtype [41, 42]. In Uganda and Zimbabwe, HIV-1 subtype D is associated with faster disease progression [43–46] but lower transmission rates than subtype A [45, 47]. Furthermore, subtype C viruses were linked with rapid disease progression in South African women [48]. The difference in disease progression between HIV-1 subtypes is likely multifactorial [33–35, 39, 46]. However, functional differences between HIV-1 subtypes may influence reservoir formation, kinetics, and the efficacy of a cure therapeutic. “HIV-1 acquisition risk (discussed extensively in [49]) and disease progression is known to differ between the sexes. Women exhibit a faster progression to AIDS-defining illness than men, even at matched viral loads [50] and have higher levels of inflammation and immune activation [51, 52] than men. In addition, women are the most affected by HIV-1 in sub-Saharan Africa and disproportionately contribute to the global burden of disease [49]. Thus, it is imperative that we tailor cure therapeutics to address an HIV-1 cure in this population.”

Predicting the effectiveness of cure strategies against different HIV-1 subtypes will be complicated. Some cure therapeutics aim to activate transcription by targeting specific HIV-1 sequences, which will likely vary between the subtypes. Patterns of recombination sites in the host genome differ by subtype [53]. Integration site may also be relevant in determining HIV-1 persistence and vary by subtype. New data shows that, compared to in vitro-derived HIV-1 integration sites, in vivo-derived sites are significantly more enriched in transcriptionally silent regions of the genome, which has relevance to reactivation of latent proviruses. With regard to subtype, integration sites from PLWH infected with HIV-1 subtype A, C or D viruses exhibited different preferences for specific genomic features and were more enriched in transcriptionally active regions of the genome compared to subtype B virus [53]. Theoretically, any type of transcriptional activation strategy to induce latency reversal may require greater potency in a subtype B setting compared to subtype A, C and D infected individuals.

### Measuring and characterizing the latent viral reservoir

The evaluation of reservoir stability and the efficacy of clinical interventions and/or changes in the reservoir after “drug holidays” or other periods of ART cessation necessitates a reliable method of quantifying the frequency of latently-infected cells that is cost and time effective [54]. This method would ideally not only enable the determination of reservoir size, but also characterize the proviral landscape to monitor qualitative changes in the reservoir. Several methods have been developed to quantify the reservoir to date. Each method measures different aspects of the reservoir and each has caveats on the conclusions that can be drawn. Table 1 provides a summary of current sizing methods and assay examples. However, only the detection of HIV-1 DNA by qRT-PCR could currently be widely implemented in most LMICs and even this methodology would be difficult to apply in some small hospitals and more rural clinics in sub-Saharan Africa. However, this method significantly overestimates the theoretical “true” replication-competent reservoir, which is the frequency of cells harbouring genetically intact proviruses that are capable of recrudescence in the absence of suppressive ART, because resting CD4^+^ T cells harbour ~ tenfold more defective than intact proviruses [55–58]. This distinction is especially important when assessing efficacy of a potential cure therapeutic, as differences in decay between intact and defective proviruses have been reported, with a slow but significant decline in intact proviruses over time on ART and little to no decline in defective proviruses [59].

**Table 1:** Methodologies for measuring the HIV-1 latent reservoir

Biomarker	Feature of the latent reservoir measured	Fraction of the reservoir included in measurement	Under/overestimation of replication competent reservoir size	Assay examples	Caveats	References
HIV-1 DNA	Either total or cell-associated HIV-1 DNA directly ex vivo by polymerase chain reaction (PCR)-based techniques	Both intact and defective HIV-1 DNA (except for IPDA); both inducible and non-induced viruses	Over	Droplet digital PCR (ddPCR); quantitative PCR (qPCR); Intact proviral DNA assay (IPDA); Q4PCR; SGS/NGS/near-full length genome sequencing (with or without integration site analysis)	With total HIV-1 DNA measurement, 2-LTR circles and episomal DNA are quantified along with proviral DNA; only IPDA and near-full length genome sequencing with integration site analysis measure intact provirus	[56, 179–182]
HIV-1 RNA induction	Cell-associated/cell-free RNA produced after ex vivo stimulation of infected cells	Both intact and defective (but transcription-competent) provirus; inducible viruses only	Over	Bulk CA-RNA PCR; Single cell RNA PCR; Single copy assay (SCA); SGS/NGS	A single round of maximal stimulation does not induce all transcription-competent proviruses	[183–188]
Induced HIV-1 protein production	Cell-associated HIV-1 proteins induced after a single round of ex vivo stimulation	Both intact and defective (but transcription-competent) provirus; inducible viruses only	Over	Flow/mass cytometry; FAST/digital microscopy; ELISA	A single round of maximal stimulation does not induce all transcription-competent proviruses; false positives associated with p24 quantitation	[189–192]
Viral outgrowth	Replication-competent virus that grows out after a single round of ex vivo stimulation of resting CD4^+^ T cells	Intact, inducible proviruses only	Under	Quantitative VOA (QVOA)	A single round of maximal stimulation does not induce all intact, inducible proviruses as some are in ‘deep’ latency	[5, 58, 60, 61]

In contrast, the quantitative viral outgrowth assay (QVOA) [5, 60, 61] is currently the gold standard for measuring the frequency of cells harbouring replication competent provirus, but underestimates the true reservoir as this assay relies on ex vivo reactivation of latently infected cells, and it has been demonstrated that in vitro reactivation methods are not 100% efficient and their efficacy may vary between populations of PLWH [58]. Additionally, this assay is resource-intensive and requires additional biosafety containment for the culture of live HIV-1. As such, the QVOA may only be practical in LMIC sites performing clinical trials of cure therapeutics and this assay would likely not be adopted as standard-of-care assay to monitor the effectiveness of cure therapeutics upon approval.

Using QVOA, the size of the latent reservoir has been estimated to be one infected cell per million resting CD4^+^ T cells in PLWH in North American cohorts [62, 63]. Historically in sub-Saharan Africa, treatment has most commonly been initiated in chronic infection, leading to an expectation that the barrier to a cure would to be greater due to lack of restriction of reservoir size through early treatment (as highlighted in the section to follow). However, we have previously reported that PLWH in Uganda (who initiated ART during chronic infection) have reduced replication competent reservoir size (three-fold lower by QVOA) compared to North Americans [8], and that Ugandan females have additionally smaller replication-competent reservoirs than males. Interestingly, despite lower QVOA outgrowth, HIV-1 DNA levels were similar between males and females [9]. Other studies report lower intracellular HIV-1 DNA in females compared to males [64, 65], highlighting important differences that may need to be considered when assessing latency reversal strategies. These studies in Uganda are two of only a handful of published studies of replication-competent reservoir size in Africans, highlighting the need for more studies in diverse LMIC settings to examine if other differences in populations exist that may help us to better understand the latent reservoir. Given that close to two thirds of women bear the burden of disease in some LMIC [66], such differences between the sexes need to be established if a global cure is to be achieved. Furthermore, only one study exists showing the association between early ART initiation and HIV-1 DNA levels in children, highlighting a paucity of cure studies in children living with HIV-1 in LMICs.

### HIV-1 reservoir establishment, heterogeneity, and kinetics

Studying the mechanisms that dictate reservoir establishment is challenging. Specifically, cells isolated from an infected individual have already entered into a latent state and therefore can only provide a pseudo-measure of this process. The long-lived reservoir in PLWH displays significant heterogeneity in sequence composition, clonality, genomic integration sites, rates of decay and the proportion of replication-competent to defective proviruses present in cells.

Infected resting memory CD4^+^ T cells are the most well-characterised of HIV-1 reservoir cells, are highly stable, with a half-life (t_1/2_) of ~ 44 months, and can theoretically persist for the lifetime of an affected individual [6, 62, 63]. Resting CD4^+^ T cells exhibit decreased expression of T cell activation markers, lower RNA content, and are not cycling [67], resulting in reduced HIV-1 transcription and favouring latency. Activated effector CD4^+^ T cells represent a primary target for HIV-1 infection due to their high permissiveness and metabolic state relative to resting cellular subsets. Many of these cells, as well as other non-HIV-specific CD4^+^ T cells, are productively infected with HIV-1 and produce viral products, which are then detectable by host immune mechanisms. Such cells are primed for elimination and are unlikely to contribute to persistence in vivo [68, 69]. Rather, latency establishment likely occurs as cells that are transitioning towards a long-lived memory phenotype [70]. Alternatively, proviral integration can occur directly in resting CD4 T cells [71, 72]. However, despite their high abundance in the body, resting cells are relatively resistant to infection, due to low expression of the HIV-1 co-receptor CCR5, limited dNTP availability, and an increase in heterochromatic structures [73, 74].

#### Differences in integration sites, viral diversity, and decay in the HIV-1 reservoir

Following transmission, HIV-1 variants diversify rapidly [75–79], reaching a plateau in chronic infection [80]. However, not all variants are equally likely to be represented in the long-lived viral reservoir [10]. Rather, variants present at the time of ART initiation are significantly over-represented, measured by both HIV-1 DNA [81, 82] and QVOA [10]. Our South African study (where subtype C predominates) evaluating cells from nine women showed that 17 to 100% (average: 71%) of the replication-competent viruses in the reservoir after five years of suppressive treatment were genetically similar to the viral variants circulating in the patient’s plasma the year immediately preceding ART initiation [10]. In comparison, the percentage of viruses seeded into the long-lived reservoir within the first year from the estimated time of infection ranged from 0 to 17% (average: 4%). Similarly, a study in Kenyan women on ART for up to 5 years showed that 59 to 99% (median: 86%; measured by *gag* sequences) of HIV-1 DNA during ART comprised of sequences present in plasma within 2 years of ART initiation [82]. These findings indicate that the long-lived reservoir may not be formed continuously at the same rate. Instead, it is possible that ART initiation establishes an environment favouring latency. Work by Jones et al. [83, 84] highlights the persistence of viral variants from earlier time-points in infection, even if in smaller proportion. The mechanism of persistence of these variants or factors affecting their longevity in the reservoir are still to be elucidated.

In early treated individuals infected with subtype C viruses, the reservoir was found to have a majority of proviral sequences intact, a low frequency of hypermutated genomes, and a paucity of truncated genomes which are found commonly in chronically-infected, untreated individuals [85]. While this scenario may not represent the majority of PLWH in LMICs due to late initiation of ART, the future of cure in these settings will benefit from more longitudinal studies characterizing reservoir heterogeneity.

#### Cellular and tissue reservoirs

One area with currently no reported characterization in LMIC studies to our knowledge, is that of cellular and tissue reservoir sites. Specific lineages of memory CD4^+^ T cells, the major cellular reservoir of HIV-1, can persist for an individual’s lifetime. Their memory differentiation status, as well as their functional polarization dictates longevity, anatomical location, and likelihood of being a stable reservoir. Central memory (T_CM_) cells are most likely to harbour provirus, followed by transitional memory (T_TM_) and effector memory (T_EM_) T cells [86–88]. A recent study showed that intact proviral DNA copies in each of these subsets varied greatly between individuals [57], but the distribution of relative abundances were similar between subsets and there were no differences in the contribution of each subset to the total pool of intact proviral copies. Less abundant subsets, such as stem cell memory T cells (T_SCM_), also contribute to the reservoir, although only substantially in some individuals [89, 90]. Early treatment appears to increase the contribution of T_SCM_ to the total CD4^+^ reservoir, reducing per-cell HIV-1 DNA levels in T_EM_ and T_TD_ (terminally differentiated) subsets [91]. The contribution of each subset to the reservoir over time on suppressive ART is difficult to study due to the dynamic nature of cell differentiation where a cell can evolve from one subset into others. Thus, the contribution of each subset to rebound virus remains an understudied topic. However, studies have showed that viral rebound likely does not have a singular source of cell type or anatomical location [92–94]. The proportion and number of T cell subsets during disease and following treatment is poorly understood between viral subtypes and may also differ between early- and late- treated populations. This merits further investigation in the context of the HIV-1 reservoir in LMICs vs. HICs.

#### Clonality and homeostatic proliferation

The proliferation of CD4^+^ T cells brought about by proliferation from homeostatic regulation and in response to antigen stimulation [95] results in large pools of clonal sequences in vivo [92, 96–102], comprising both defective and intact proviruses. In our South African study, we reported between 0 and 47% (by sequence identity) of outgrowth viruses form PBMCs were clonal in nature [10]. Furthermore, another small study found that clonal sequences were rare over the first year of ART in early treated PLWH, providing insights into the difference in reservoir composition between early- and late-treated individuals infected with subtype C viruses [85]. Replication-competent clonal populations have been found to be distributed across different T cell memory subsets [57, 92, 103], indicating that infected cells differentiate and proliferate during ART unabated by the immune system [103, 104]. In addition to memory differentiation, differences in functional polarization may influence reservoir size. One study found that functional polarization may lead to preferential clonal expansion of replication-competent HIV-1 in Th_1_ cells [105]. Furthermore, these clonal populations are found in different tissues and can contribute to plasma viral rebound when treatment ceases [92, 94]. A caveat with some of these studies is that frequently only a small region of the HIV-1 genome is sequenced, so that it cannot be definitively known if cells with identical sequences in that region are the result of integration by a pool of homogenous viruses, or due to homeostatic proliferation of infected cells, the latter defining a true clonal population. Integration site analysis is required to distinguish between these two possibilities, as the likelihood that integration will occur multiple times in the same location in the human genome is negligible [106]. Nevertheless, it is clear that clonal populations of HIV-1 contribute markedly to reservoir maintenance. Antigenic stimulation and immune modulation are likely different in LMICs vs. HICs, and further studies are needed to assess the contribution of clonal expansion and latency in different cellular subsets in other populations in LMICs.

### Viral factors contributing to HIV-1 persistence

The role of infecting subtype in reservoir formation, size and maintenance is greatly understudied. Particularly in LMIC where the predominant infecting subtypes differ from the most studied cohorts (predominated by subtype B infections), this may result in significant geographical differences in the latent viral reservoir. The viral promoter element of HIV-1, the long terminal repeat (LTR), has been reported to impact latency [107], with ‘latency potential’ (defined as the ratio of latently infected cells to actively infected) differing between subtype-specific LTR genotypes in vitro [108]. In subtype C viruses, increased levels of transcriptional activity resulted in more rapid silencing of the viral promoter due to negative feedback, and was associated with a greater number of NF-ĸB binding sites within the LTR [109]. Furthermore, the AP-1genotype (a binding motif in the LTR) has been shown to confer greater latency potential (i.e. reduced proportion of cells that lack transcription of HIV-1 genes) to subtype A and C viruses compared to subtype B [110]. Conversely, another study showed no differences in initial latency potential between subtypes with the exception of subtype AE in primary T cells [111].

The HIV-1 transcriptional switch protein, Tat, is heavily implicated in the establishment and persistence of latent provirus. Tat regulates transcriptional elongation via RNA polymerase II (RNAP II) recruitment in the viral 5’ LTR. For viral transcription to occur, Tat must bind the trans-activating response (TAR) hairpin present on the viral transcript. In a study by Razooky et al*.*, Tat-mediated feedback was able to induce proviral reactivity in the absence of cellular stimulation [112]. In fact, in the absence of multiply spliced RNA encoding *tat/rev*, the feedback loop was disrupted and led to non-productive infection [113]. Due to the heavy implication of HIV-1 Tat on transcriptional status, this protein is critical to many therapeutic strategies being utilized today, highlighting the importance of characterizing *tat* variants in the reservoir. Subtype C Tat (TatC) has been shown to have a higher transcriptional activity in T cell lines than Tat from subtypes B (TatB) and E [114]. Furthermore, genetic variations in TAR can impact the ability of Tat to facilitate viral transcription [115]. Studies identified intra-subtype C variation in the TAR element as well as Tat that correspond to key functional sites that affect Tat binding and Tat-induced transcriptional activity, respectively [116–118]; including evidence of positive selection in primary infection [117]. In addition to mediating transcription, the viral protein Tat is also responsible for RNA silencing suppressor activity (RSS) in infected cells [119–122]. RSS serves to attenuate translation of HIV-1 transcripts, determining viral load set-point and favouring latency. TatB has been shown to have more potent RSS activity than that of TatC viruses, however, a greater range of RSS activity was observed among TatCs [123].

The HIV-1 accessory protein Nef is an element of interest as a reservoir determinant. Nef facilitates the pathogenesis of HIV-1 by interfering with host protein trafficking [124]. Furthermore, sequestration of major histocompatibility complex-1 (MHC-I) by Nef precludes antigen presentation by infected cells and evasion of the host CTL response as a result [125, 126]. In a recent study, the strain-specific ability of Nef to downregulate MHC-I in vitro was associated with in vivo reservoir size [127]. Furthermore, in a multivariable analysis adjusting for multiple clinical factors, HIV-1 DNA levels were found to be higher in individuals infected with subtype B compared to those infected with non-B subtypes (CRF_01 AE and G), and this higher abundance of cells harbouring HIV-1 DNA was attributable to the superior Nef function of subtype B viruses. However, this study included only men who initiated treatment in acute/early infection and were on treatment for less than a year [127]. Given that Nef function has been shown to differ across subtypes, with subtype C exhibiting reduced Nef function compared to other subtypes [128], it will be beneficial to assess Nef function in the context of other HIV-1 subtypes, females, and PLWH who initiated treatment during chronic infection, to determine the generalizability of these findings to LMIC.

### Clinical and immunological correlates of the HIV-1 reservoir

The most well-characterized clinical measure that correlates with reservoir size is pre-ART viral load (VL). Several studies show that pre-ART VL setpoint, or even just the VL the time of ART initiation, correlates positively with HIV-1 DNA [13, 127] and QVOA estimates of reservoir size [8, 9], which is consistent with studies showing that early ART restricts reservoir size (both replication-competent and HIV-1 DNA) [91, 129–131]. In ART-treated patients, CD4 counts over time have also been shown to predict replication-competent reservoir size in PLWH who initiated treatment in acute infection [132], and several studies have identified the extent of CD4 depletion as shaping the HIV-1 DNA proviral load [133, 134], with nadir CD4 count and CD4:CD8 ratio shown to be strong negative correlates of reservoir size. Furthermore, time on ART has also been shown to correlate negatively with replication-competent reservoir size [9].

Events soon after the establishment of infection impact disease progression, and there is mounting evidence that this may be the case for characteristics of the latent reservoir. Immune activation and inflammation play an important role in the disease progression of HIV-1 [135, 136]. Immune activation is very strongly correlated with set-point VL, and predicts progression to AIDS-defining illness more robustly than VL [13, 135, 137]. Early initiation of ART not only reduces the cumulative viral burden before ART, preserves CD4^+^ T cells, and maintains CD4:CD8 ratios, but also reduces T cell activation and inflammation [11–13]. T cell activation may increase the pool of target cells available to sustain HIV-1 replication, but also augments antigen-driven clearance of infected cells and thus increases T cell turnover. Following ART initiation, many measures of immune activation decline rapidly, but this does not occur equally in all individuals, and residual inflammation is a strong predictor of non-AIDS mortality in the context of successful ART [138]. Understanding upstream contributors to T cell activation, such as cytokine expression throughout both treated and untreated infection, may shed light on subsequent HIV-1 reservoir dynamics over time. IL-10, and sTNFRII concentrations were positively associated with levels of total HIV-1 DNA in peripheral blood mononuclear cells (PBMCs) after 96 weeks of treatment [13], while MIP-3β showed a trend towards a correlation.

Since immune activation and inflammation are known to differ between the sexes, detailed studies into the immune correlates of reservoir size in males and females are needed. We recently reported a difference in the immune correlates between males and females in PLWH in Uganda [9]. While in males the frequency of PD-1^+^ CD4^+^ T cells and IL-2^+^ CD8^+^ T cells were positive and negative correlates of replication-competent reservoir size, respectively, only TNF^+^ CD8^+^ T cells was found to have a positive association with replication-competent reservoir size in females. The lack of a positive association between PD-1 expression and reservoir size in women may be of particular concern, as this association, well-established in men, has led to the development of PD-1 agonists as latency reversal agents. The efficacy of this therapy, and other immune-based cure therapeutics, should be carefully tested in both sexes.

Several studies have suggested that inflammation and immune activation are different in African cohorts [139–143]. High levels of genital inflammation have been observed in a well-characterized cohort of South African females [144] and high levels of inflammation during early infection in this cohort correlated with VL set-point and, to a lesser degree, disease progression (as measured by CD4 depletion). Few studies have addressed ongoing immune activation in the context of Africans receiving ART. Understanding of the impact of immune activation on viral reservoir dynamics at multiple stages of HIV-1 infection, particularly in cohorts where ART was initiated in chronic infection, is imperative to formulating cure strategies in LMIC.

Finally, the paucity of reservoir studies in different geographical settings also means that critical information on reservoir establishment and dynamics in the face of co-infections is overlooked. Moreover, an in-depth discussion of co-infections and the HIV- 1 reservoir is precluded by the absence of studies on the topic. Co-infections have been shown to increase pathogenesis and HIV-1 viral loads, increase immune activation, and inflammation. Thus, our discussion on how increased or decreased viral loads, immune activation, and inflammation impacts reservoir size would extend to the more general effect of co-infections. Effective treatment of co-infections (excluding hepatitis B and C) results in a reduction of plasma viraemia in ART naïve PLWH [1]. As there remains fundamental questions on the timing of viral reservoir seeding prior to ART initiation [10, 145–148], the effects of longer term (e.g. TB) or periodic/sporadic (e.g. malaria or HSV) co-infections on the viral reservoir may depend on the timing of these event(s) during acute and chronic disease and the timing of ART initiation. Furthermore, infections that lower the barrier to HIV-1 acquisition such as bacterial vaginosis and other sexually transmitted infections, resulting in an increased HIV-1 risk or a higher multiplicity of infection, may influence HIV-1 reservoir size or composition. This topic has been reviewed extensively elsewhere [2], including a detailed summary of knowledge gaps with regards to specific co-infections such as Mycobacterium tuberculosis, Hepatitis B and C, helminth infections, and other STIs. LMICs have a higher burden of co-infections and thus, latent reservoir studies in this context are of great importance for cure strategies in the future.

### Strategies for HIV-1 eradication and cure

In the context of HIV-1, researchers often refer to cure as either ‘functional’ or ‘sterilizing’. In either instance, a cure would allow for PLWH to interrupt ART without experiencing viral rebound. For a functional cure, HIV-1 is durably controlled in the absence of ART, while remaining, in some form, within the body. Alternatively, a sterilizing cure aims to remove all traces of HIV-1, including provirus, from PLWH. The primary strategies being investigated for HIV-1 cure are mentioned below and will highlight the practicality of implementation of these strategies/therapeutics in LMICs based on ease of potential clinical use, cost, and availability.

#### Hematopoietic stem cell transplant

To date, an apparently sterilizing cure has been achieved in two individuals [149, 150]. These individuals received a homozygous CCR5∆32 hematopoietic stem cell transplant following immune ablation. The CCR5∆32 mutation renders cells impervious to strains of HIV-1 that use CCR5 as a co-receptor. These; the ablation eliminates a substantial portion of the cells harbouring replication competent provirus, and the transplanted CCR5∆32 that reconstitute the immune system are resistant to HIV-1 infection. While these cases of sterilizing cure offer proof that a cure is possible, this procedure is associated with high mortality and would not be appropriate for an otherwise healthy person living with HIV. For those with treatment access, ART provides a lower risk alternative with relatively fewer co-morbidities, while excluding the possibility of treatment-associated death. Additionally, the high procedure-associated cost (i.e., approaching several hundred thousand dollars per patient for treatment, hospitalization, and follow-up monitoring) limit scalability and global rollout. Aside from the risk to the patients, this approach will never be widely adopted even in HICs based on cost alone.

#### Shock-and-kill

The ‘shock-and-kill’ strategy aims to induce transcriptional reactivation of the replication-competent proviral reservoir, and has been recently reviewed by Kim et al. [151]. The intention is that induced cells will produce viral products that are recognizable by host immune mechanisms, prompting their clearance by cytotoxic immune mechanisms. During this process, an individual would maintain daily ART to prevent de novo infection of bystander cells by virus produced by reactivated cells. Since post-ART rebound occurs at a frequency of approximately once a week, a substantial proportion of the reservoir must be cleared for this approach to be feasible [152]. For instance, a 1,000-fold reduction in the replication competent reservoir is theoretically required to achieve an average remission of 20 years [153]. Such modeling reveals the innate challenge of developing a functional ‘shock-and-kill’ therapeutic, while also indicating that it is theoretically possible with the correct approach.

Latency-reversing agents (LRAs) comprise small molecules and biologics designed to induce transcriptional reactivation of virally infected cells. Amongst the most investigated are histone deacetylase inhibitors (HDACi), which function through direct inhibition of the HDAC enzyme. HDACi can induce highly variable latency reversal in cell lines engineered with latent HIV-1 provirus [154] but fail to induce appreciable HIV-1 from cells isolated from PLWH on stable ART [155–157]. HDACi activate a low level of transcription, but undetectable HIV-1 protein synthesis, which is necessary for immune recognition and elimination of these latently infected cells. They have been utilized with low level, daily dosing in phase I/II clinical trials, but have failed to reduce the latent HIV-1 pool [145]. Increased dosage may provide better latency reversal, but HDACi are not selective for latent, integrated HIV-1 proviral DNA and would result in general transcription upregulation of host genes.

In lieu of epigenetic manipulation, some approaches target cellular activation pathways. Protein kinase C (PKC) agonists, for example, induce global T cell signalling and transcription factor recruitment [158]. Similarly, toll-like receptors (TLRs) that recognize RNA viruses, such as TLR 3/7/8, can mediate T cell activation and downstream induction of latent proviral expression, while also enhancing cytolytic activity [159]. One example is the TLR-7 agonist, GS-9620 (Vesatolimod; Gilead), which became a strong candidate after pre-clinical data showed clearance of hepatitis B in several models of infection [160, 161]. In clinical trials, GS-9620 could potently induce expression of interferon-stimulated genes (ISG), leading researchers to investigate the agonist’s potential as a latency-reversing agent for HIV-1. A subsequent ex vivo study revealed that TLR-7 agonists could promote HIV-associated RNA production by 1.5–twofold [162]. In a recent Phase Ib study, GS-9620 was well-tolerated at doses sufficient to induce ISG expression (> 4 mg) [163], providing feasibility for use in future regimens.

Interestingly, several studies have indicated that optimal latency reversal could require stimulation through the T cell receptor (TCR) [164, 165]. Signalling through the TCR, best demonstrated by the use of PMA/Ionomycin or anti-CD3/anti-CD28 antibodies, results in an intracellular cascade for multi-kinase activation, chromatin remodelling and transcription factor induction necessary for immune activation of memory and naïve T cell subset [166]. Interestingly, the same activation cascade is required for productive HIV-1 replication in activated T cells, and reactivation of latent provirus is more potent after stimulation with HIV-1 antigen, as opposed to non-HIV-specific antigen, which may be due to the preferential infection of HIV-specific T cells [167]. Upon primary HIV-1 infection, antigens consisting of HIV-1 virus particles and proteins are transported to primary and secondary lymphoid tissues/organs by antigen presenting cells. These APCs will ultimately present HIV-1 antigens on MHC class II to CD4^+^ T cells with TCR specific to these HIV-1 antigens, leading to activated CD4^+^ T cells, which are now susceptible to HIV-1 infection and replication [168]. We hypothesize that this cycle of HIV-1 antigen presentation and activation of HIV-specific T cells leads to a skewing of the antigen-specificity of latently infected cells towards HIV-1 antigens [169]. This hypothesis is supported by the observation that most variants in the reservoir arose from strains circulating immediately prior to ART initiation [10, 81, 82]: once ART is initiated and HIV-1 antigens are cleared HIV-specific T cells will revert to a resting state *en masse*. The use of HIV-1 antigens as an immunogen could specifically reactivate and eliminate this substantial portion of the reservoir [169], We have recently developed heterogenous virus-like particle (VLP) derived from the quasi-species of five HIV-1 infected patients [169, 170]. This VLP formulation contains all the HIV-1 proteins and is morphologically identical to wild type HIV-1 but lacks genomic RNA and has additional mutations to ensure it is not replication-competent and can be tested as a cure therapeutic [170]. Heterogenous HIV-1 VLPs have so far out-performed all other LRAs at induction of the latent HIV-1 pool from CD4^+^ T cells isolated from patients on stable ART. The level of latent HIV-1 induction by these VLPs is comparable to PMA/ionomycin but the VLPs, unlike PMA/ionomycin, target only a fraction of CD4^+^ T cells for activation and therefore is unlikely to create generalize and toxic immune activation. These findings suggest that VLPs may result in targeted activation of a sizeable proportion of the latent pool. However, for this therapeutic to be useful in LMIC, the effect of subtype congruency between VLP and patient must be ascertained. Additionally, while activation of the latent HIV-1 pool in response to VLP has been observed using cells form patients treated during both acute/early [169] and chronic infection, it must be ascertained if the reservoir of individuals treated during chronic infection is as heavily skewed towards HIV-specific T cells. Individuals who initiated ART soon after infection will have been exposed to fewer non-HIV antigens while viremic, but only a minority of PLWH in LMIC initiated treatment during acute infection. Aside from these remaining uncertainties, compared to the use of small molecules lacking a specific targeting mechanism, the use of VLPs is safe, effective, and is an inexpensive cure therapeutic that could potentially be widely used around the world.

Many of these small molecule or even peptide-based inhibitors/agonists would be a suitable cure therapeutic for testing and potential roll-out in LMICs. Importantly, such strategies would greatly benefit from longitudinal follow-ups to ensure viremia is suppressed in the absence of ART. Currently, their cost remains prohibitive, but with relaxation of the agreement on trade-related aspects of intellectual property rights (TRIPS) agreement/patent regulations for greater drug access for LMIC markets, it is possible that investment by the Global Fund, PEPFAR, and various other foundations, would be offset by the reduction in the burden of ART, the concern of ART drug resistance, and the prevention of new infections.

#### Block-and-lock

The ‘block-and-lock’ approach aims to provide a functional cure for HIV-1 through the suppression of transcriptional activity. One way to achieve this is by altering methylation and acetylation status. For a block-and-lock approach, this will typically involve a combination of hyper-methylation and/or hypo-acetylation. Furthermore, many strategies are utilizing small interfering (si) RNA to induce epigenetic changes at sites of transcriptional relevance, such as the HIV-1 NF-kB promoter site [171–173]. An alternative approach is blocking the HIV-1 accessory protein, Tat, which is involved in the recruitment of RNA polymerase II. Blocking its function might greatly limit transcriptional output. Didehydro-Cortistatin A (dCA) is one small molecule capable of engaging with HIV-1 TAR RNA—the binding domain of Tat [174]—and dCA treatment renders cells resistant to the effects of LRAs [175]. Another study proposed the use of “naked” cyclic Tat peptidomimetics that have rapid cellular uptake, that bind HIV-1 TAR RNA with high affinity, and inhibit Tat transactivation/mRNA transcription as well as reverse transcription [176, 177].

For a block-and-lock strategy to be curative, virtually all replication competent provirus would require durable, drug-mediated post-translational modification. This means that, even if a small proportion remains unmodified, the chance of productive re-infection exists. However, studies have shown that reducing the size of the replication competent reservoir can prolong the time to viral recrudescence [153]. This would arguably be true of epigenetic silencing as well. Therefore, the lock-and-block strategy likely reduces risk of re-infection short-term, but will invariably lead to rebound unless complete, sustained reservoir silencing is achieved.

The block-and-lock approach is uniquely positioned from a therapeutic perspective. Since the goal here is not the removal of the viral genomes but rather their suppression, blocking agents could potentially function on multiple distinct cellular reservoirs. Block-and-lock inhibitors are also easier to design, test, and possibly utilize for treatment. However, long acting formulations are necessary for these inhibitors to be considered as a cure therapeutic, as opposed to a form of ART. Long-acting ART will provide a benefit for those PLWH who have issues with adherence (adolescents, etc.), and was found to be more acceptable than oral ART in the context of pre-exposure prophylaxis [178], but may not replace oral ART completely in the very near future. One of the drawbacks of long-acting ART administration is having more frequent clinic visits: once a month or every two months as opposed to biannually. In addition, well-resourced settings may be able to handle the operational challenges associated with switching from oral to injectable medications: potentially more staff required on site, more frequent VL testing to ensure viral suppression between visits, increased challenges with regards to ensuring all visits occur timeously, storage and transport of injectables/infusions. In terms of LMICs, this may ease the burden on PLWH who have access to these resources in urban and suburban settings but may not work well in rural settings unless distribution of these interventions can be decentralized (i.e. administered outside of a clinical setting). More research is needed into whether these treatments can be administered by non-medical personnel or whether self-administration of these drugs is feasible. Despite the challenges, intermittent administration of block-and-lock therapeutics could reduce pill burden and alleviate ART-associated co-morbidities. As with all potential therapeutics, global investment and robust organizational initiatives are mandatory for proper roll-out and maintenance in LMICs, assuming a clinically successful ‘block-and-lock’ strategy is realized.

#### Broadly neutralizing antibodies as a cure therapeutic

The discovery of monoclonal antibodies capable of potent neutralization of a wide range of HIV-1 isolates, termed broadly neutralizing antibodies (bNAbs), has provided new avenues for functional cure research. In-human trials (passive immunization) have been conducted with several bNAbs and they have been shown to be tolerable and safe, even at high doses and when administered repeatedly in both HIV-1 uninfected [4, 5] and infected adults [6, 7]. Monoclonal bNAb therapy did not result in decrease in residual plasma viraemia [8], HIV-1 DNA/RNA [8, 9] or outgrowth viruses (demonstrated with VRC01) [8] but did result in a delay in viral rebound after analytical treatment interruption (ATI), demonstrated with VRC01 [10, 11], 3BNC117 [12], and UB-241 [13], although results were highly variable across trials (ranging from 4 to 16 weeks median time to rebound). Combination bNAb therapy has also shown promise, with the benefit of a decreased likelihood of developing resistance to multiple antibodies [7]. Trials testing sequential administration of 2G12, 2F5, and 4E10 resulted in 8–10-week delay in viral rebound upon ATI [14, 15]. Administration of 3BNC117 and 10–1074 in combination during ATI resulted in maintained viral suppression for a median of 21 weeks in participants who had antibody sensitive viral reservoirs [6].

A phase I clinical trial is ongoing in South African women (CAPRISA 012), testing CAP256V2LS, a potent bNAb isolated from a subtype C infected individual capable, alone and in combination with VRC07-523LS and PGT121 for use in pre-exposure prophylaxis [16]. However, this trial could pave the way for investigation into CAP256V2LS (likely in combination) as a functional cure in Africans. Finally, the first studies in non-human primates showed efficacy of bNAbs produced in vivo through the administration of adeno-associated viruses encoding bNAbs [17]. Although host-elicited immune responses limited effectiveness of the treatment [18], one macaque was functionally cured. While this approach is still in development and needs refining before in-human trials, it is a proof of concept for the role bNAbs in a functional cure strategy. A disadvantage associated with bNAb therapy in resource-limited settings is the requirement for resistance screening both before administration of therapy to ensure antibody sensitivity of viruses, and during therapy to monitor antibody escape. Similar to long-acting ART, these drawbacks significantly limit the scalability of bNAbs as a cure therapeutic.

## Summary

The development of a HIV-1 cure has been a major initiative for the global scientific community and a dominant target for drug development. Having over 40 million people on lifelong ART is not a sustainable global strategy and with increasing circulation of drug resistant HIV-1, new infections may not be prevented by ART alone. An affordable and effective cure therapeutic that could be rapidly deployed during first ART is a necessary goal for the elimination of HIV-1 from the human population. However, HIV-1 cure research has been largely focused on the eventual application to PLWH in HICs, and potential therapeutics are not being designed or tested with the majority of PLWH in mind. Until the rollout of second-generation INSTIs for treatment, the rate of treatment failure and resistance was already preventing the testing of cure therapeutics in as much as 10–20% of the 20 + million PLWH receiving ART in LMICs. Considering HIV cure strategies/therapeutics currently under investigation, cost and feasibility present challenges to implementation in LMICs. This is complicated by the lack of studies characterising reservoir size, composition and determinants of HIV persistence in these key populations. We identified research topics that would aid in the development and implementation of a global cure therapeutic: (i) reservoir sizing studies in LMICs and the development of a cheap, meaningful and standardized assay to assess reservoir size changes in PLWH; (ii) identifying subtype differences in various HIV-1 proteins/elements linked to pathogenesis including Tat, the LTR, and Nef, along with their interplay in reservoir establishment and kinetics; (iii) differences in clonality and cellular distribution of the reservoir particularly in chronically-treated individuals. Finally, resources need to be made available for the study of cure therapeutics in the setting of LMICs, since this will address the largest burden of HIV-1 infection globally.

## Data Availability

Not applicable.

## Abbreviations

PLWH: People living with HIV-1
ART: Antiretroviral therapy
LMICs: Low- and- middle-income countries
HICs: High income countries
HIV-1: Human immunodeficiency virus type 1
VL(s): Viral load(s)
PEPFAR: U.S. President’s Emergency Plan for AIDS Relief
NRTI (s): Nucleoside reserves transcriptase inhibitor(s)
NNRTI(s): Non-nucleoside reserves transcriptase inhibitor(s)
EFV: Efavirenz
AZT: Zidovudine
TDF: Tenofovir disoproxil fumarate
3TC: Lamivudine
PI(s): Protease inhibitors
DTV: Dolutegravir
FTC: Emtricitabine
QVOA: Quantitative viral outgrowth assay
LTR: Long terminal repeat
TatB: HIV-1 subtype B Tat protein
TatC: HIV-1 subtype C Tat protein
MHC-1: Major histocompatibility complex 1
T_CM_: Central memory T cells
T_TM_: Transitional memory T cells
T_EM_: Effector memory T cells
T_SCM_: Stem cell-like memory T cells
T_TD_: Terminally differentiated T cells
PBMCs: Peripheral blood mononuclear cells
T_h_: Helper T cell
LRAs: Latency-reversing agents
HDACi: Histone deacetylase inhibitors
HMTi: Histone methyltransferase inhibitors
DNMTi: DNA methyltransferase inhibitors
TLR: Toll-like receptor
TCR (s): T cell receptor(s)
VLP: Virus-like particle
dCA: Didehydro-cortistatin A
